# Superior Disembedding in Children with ASD: New Tests Using Abstract, Meaningful, and 3D Contexts

**DOI:** 10.1007/s10803-018-3508-y

**Published:** 2018-02-21

**Authors:** Ruth Van der Hallen, Rebecca Chamberlain, Lee de-Wit, Johan Wagemans

**Affiliations:** 1 Laboratory of Experimental Psychology, Department of Brain and Cognition, KU Leuven, Leuven, Belgium; 2 Leuven Autism Research (LAuRes), KU Leuven, Leuven, Belgium; 3 Clinical Psychology, Erasmus University Rotterdam, Rotterdam, The Netherlands; 4 Department of Psychology, Goldsmiths, University of London, London, UK; 5 Cognition and Language Sciences, Chandler House, University College London, London, UK

**Keywords:** Autism spectrum disorder (ASD), EFT, L-EFT, Disembedding, Perceptual organization

## Abstract

Since its initial development, the embedded figures test (EFT) has been used extensively to measure local–global perceptual style. However, little is known about the perceptual factors that influence target detection. The current study aimed to investigate *disembedding* in children with and without ASD, aged 8–15 years, using the newly developed, stimulus-controlled L-EFT, M-EFT and D-EFT. Firstly, results revealed superior disembedding for children with ASD, irrespective of the type of target or embedding context, although the ASD group took more time in both the M-EFT and D-EFT. Secondly, the number of target lines continuing into the context proved more of a hindrance for the controls. Taken together, these findings provide strong evidence to support the notion of superior disembedding in ASD.

## Introduction

Autism spectrum disorder (ASD) is a neurodevelopmental condition, affecting approximately 1% of the population. It is best characterized by deficits in social communication and interaction, as well as restricted, repetitive patterns of behavior or interest, including atypical responses to sensory input or unusual interest in sensory aspects of the environment (DSM 5; American Psychiatric Association 2013). For over three decades, researchers have been investigating to what extent individuals with ASD present with atypical perceptual organization compared to typically developing (TD) individuals (Evers et al. 2018).

Perceptual organization, as defined by Palmer (1999), is “the process by which the bits and pieces of visual information that are available at the retinal image are structured into the larger units of perceived objects and their interrelations” (p. 255). As a result, the incoming sensory information does not appear to us as a collection of disjointed sensations, but gives rise to a particular organization of spontaneously combined and segregated objects (Wagemans 2018; Wagemans et al. 2012). The main prerogative is that one will *see the forest before the trees*, and be able to discern the overall pattern or global picture first, prior to perceiving the underlying mass of individual elements or details.

Individual differences in perceptual organization have long been ignored in the majority of studies. In the last decade or so, however, it has become an important topic of research (for a recent overview, see de-Wit and Wagemans 2015). The working assumption is that all individuals are characterized by a distinct perceptual profile, with variable degrees of a more global or a more local perceptual bias, notwithstanding our general tendency to *see* in terms of the wholes rather than the parts. Such individual differences, influencing perceptual and cognitive functioning, have been revealed with regard to expertise, culture and psychopathologies. For instance, researchers have revealed enhanced local visual processing in artists and musicians (Chamberlain et al. 2013; Drake and Winner 2011; Stoesz et al. 2007) or a reduction in global bias in remote cultures a result of reduced exposure to urbanized environments (Caparos et al. 2012).

Shah and Frith (1983, 1993) were the first to publish results suggestive of a distinct perceptual profile for individuals with ASD, namely enhanced local and reduced global visual processing in individuals with ASD. Ever since Shah and Frith put this idea forward, the interest in perceptual organization in individuals with ASD has grown tremendously (for a review, see Simmons et al. 2009). Unfortunately, evidence remains mixed and results often seem to contradict each other. The most common visuo-spatial paradigms used to investigate atypical local–global visual processing in individuals with ASD rely on the use of hierarchical letters or figures, block designs, visual illusions, or embedded figures tests.

The original embedded figures test (EFT) was developed by Witkin et al. (1971). The test consists of cards depicting images made up of lines in which simple geometrical shapes are embedded, which the participant is asked to locate as quickly as possible. The target shapes become difficult to detect by incorporating them in an embedding context that forms a strong configuration. As a result, the configuration (or whole Gestalt) tends to dominate perception and the target shape seems to be hidden or become “embedded” within the context (Goodenough and Witkin 1977). Witkin proposed the EFT as a measure of field-(in)dependence, terminology he used to refer to individuals who would present with a strong global or local bias (i.e., perception of an attribute or element *dependent on* or *independent from* the field around it, resp.) (Witkin et al. 1962). The better one performs on the EFT (i.e., faster or more accurate disembedding), the more one qualifies as field-independent, as good performance indicates that contextual information can be discounted in order to focus on the local elements of the visual field. Since its initial development, Witkin’s EFT (1971).) has been used extensively in research on individual differences, particularly in the study of local vs. global perceptual styles, both with regard to typical development and different clinical populations (e.g., Cribb et al. 2016; de-Wit and Wagemans 2015; Milne and Szczerbinski 2009; Panton et al. 2016). Adapted versions of the EFT for preschool children were made available as well (i.e., the Children Embedded Figures Test or C-EFT; Karp and Konstadt 1963).

While past use of the EFT and other visuo-spatial paradigms seems to suggest the EFT is a good paradigm to measure individual differences in local–global visual processing, intelligence, or executive functioning (Goodenough and Karp 1961; Richardson and Turner 2000; Roberge and Flexer 1981), recent research has indicated that *disembedding*, typically measured by the EFT, constitutes an independent factor or ability. In 2009, Milne and Sczcerbinski published an extensive investigation of the factorial structure of individual differences in local and global processing. Their vast set of tasks, administered to 90 TD individuals, included the Group Embedded Figures Test (G-EFT), a block design task, a hidden patterns test, a Gestalt completion test, a copying test, the VSOP silhouettes, a spot-the-difference task, the Rey Osterrieth Complex Figure, a Navon task, the Muller-Lyer illusion, a visual search task, several Kanizsa illusory surfaces and impossible figures, the good form test, and a global coherent form and motion task. Interestingly, their inter-task correlation matrix revealed a surprisingly diffuse pattern of correlations and a factor analysis revealed no more than two distinct, meaningful factors: a *disembedding* factor (which included the block design task and the G-EFT) and a *global bias* factor (which included two distinct conditions of the Navon task). From this, the authors concluded that the construct of local and global visual processing may be marred by conceptual and terminological inconsistencies. In contradiction with the prevailing assumption that all so-called local–global paradigms are measuring one and the same construct, their results showed very little common variance within the set, expect for the two distinct factors, the *disembedding* and *global bias* factor, that were revealed by the factor analysis.

More recently, additional questions have been raised with regard to the EFT stimulus sets (and its variants like the C-EFT or the G-EFT). Although popular in use, very little is actually known about the perceptual principles that underlie these stimulus sets, and, unsurprisingly, stimulus control is lacking. Visual inspection of Witkin’s embedded figures suggests that the target shapes were embedded within the context, while keeping a number of perceptual factors such as closure or symmetry in mind. However, the actual factors used to embed the target shapes were not explicitly manipulated nor discussed, rendering it unclear to what extent and in what way different perceptual factors may influence the perceived embedding. To remediate these problems, de-Wit and colleagues developed the Leuven-Embedded Figures Test (L-EFT; de-Wit et al. 2017) a well-controlled and parameterized stimulus set, made freely available to others. The L-EFT manipulates a number of factors pertaining to the target shape (i.e., complexity, symmetry and closure), while also controlling the embedding context (i.e., manipulating the number of continued target-lines, controlling the total number of lines present in the context). Manipulating the number of continued target-lines in the context or degree of good continuation, arguably the most essential manipulation of the set, was motivated by Rao and Ballard’s (1999) predictive coding account of *end-stopping*. End-stopping is a property of particular neurons in the primary visual cortex that fire in response to an edge ending at a particular point in space corresponding to the cell’s receptive field (and will not fire when that edge is continued into a longer line). The suggestion is that the detectability of individual line segments in the EFT (and the perceptual availability of those line segments to form the target shape) is reduced when these are seen as belonging to longer lines (i.e., those in the embedding context).

**Fig. 1:**
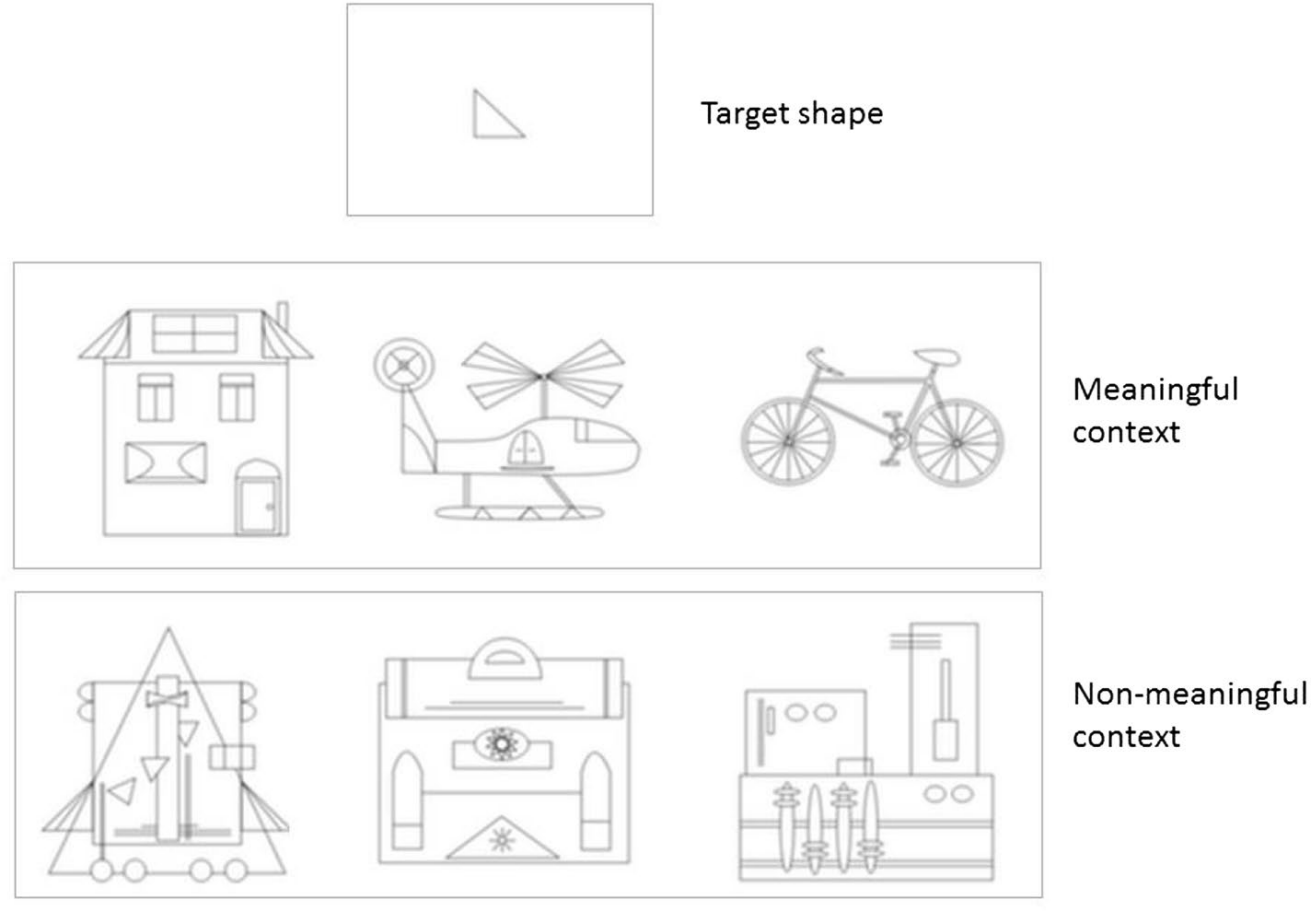
Example trial of M-EFT with meaningful and non-meaningful contexts (only one of both contexts shown in each actual trial). The correct answer is the context presented on the left (randomized in the actual trials)

**Fig. 2:**
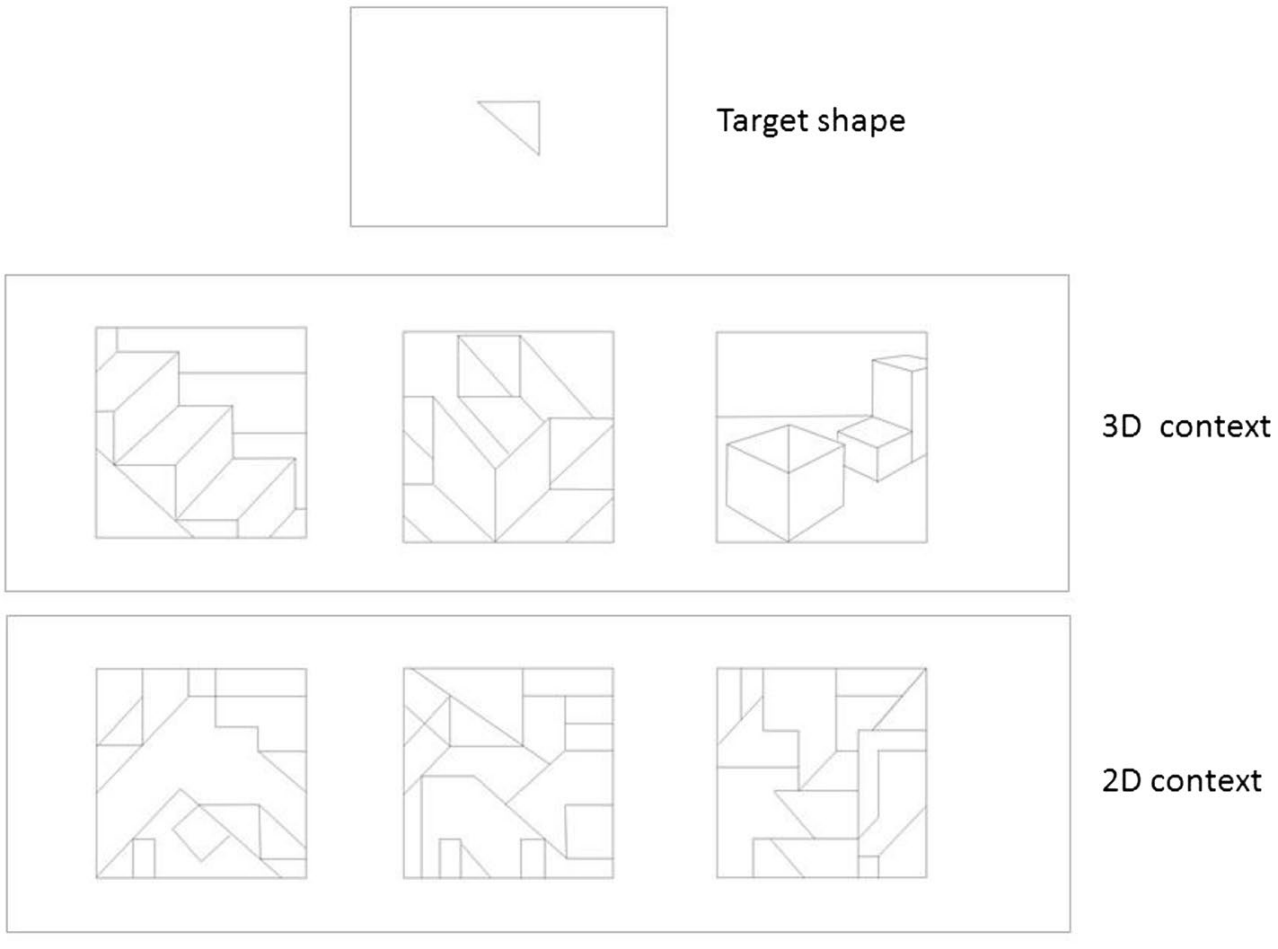
Example trial of D-EFT with 3D and 2D contexts (only one of both contexts shown in each actual trial). The correct answer is the context presented on the left (randomized in the actual trials)

In addition to the L-EFT, which comprises the core set of EFT stimuli, de-Wit and colleagues developed two modified versions each focusing on a particular aspect related to EFT, the meaningful-embedded figures test (M-EFT) and the Three-dimensional embedded figures test (D-EFT). The M-EFT is an adaptation of the L-EFT in which the embedding contexts within which participants must locate a target shape are either meaningful (they represent real objects) or non-meaningful (they represent nonsense objects composed of the same parts as the meaningful objects, see Fig. 1). The D-EFT is an adaptation of the L-EFT in which the embedding contexts within which participants must locate a target shape are either rendered in a 2D or 3D manner (see Fig. 2). Previous research has validated all three tasks, as well as their test–retest reliability, administering all three tasks to large groups of TD individuals (and in part, to individuals with distinct drawing expertise) (Chamberlain et al. 2017; Chamberlain and Wagemans 2015; de-Wit et al. 2017; Huygelier et al. 2018). However, none of these three tasks have yet been evaluated in relation to disembedding abilities in individuals with ASD.

The ability to disembed visual information or focus on local aspects of the visual field regardless of the (global) context within which it is embedded has been widely investigated in individuals with ASD using the using the EFT, C-EFT or G-EFT (for recent meta-analyses, see Muth et al. 2014; Van der Hallen et al. 2015). Of all popular visuo-spatial tasks, the EFT has gained interest due to its potential to reveal *superior* performance (rather than *impaired* performance) for individuals with ASD. However, studies using EFT to test local–global visual processing in individuals with ASD have produced mixed results, either revealing similar (e.g., Bölte et al. 2007; Brian and Bryson 1996; Chen et al. 2008; Damarla et al. 2010; Edgin and Pennington 2005; Spencer et al. 2012), enhanced (e.g., Brosnanet al. 2012; de Jonge et al. 2006; Falter et al. 2008) or diminished (e.g., Burnette et al. 2005; Edgin and Pennington 2005) performance for individuals with ASD compared to TD individuals, and/or atypical activation patterns using imaging techniques (e.g., Damarla et al. 2010; Spencer et al. 2012). All previous investigations have used the EFT, C-EFT, G-EFT or a personal, slightly modified EFT, meaning none have used an embedded figures test of which the stimulus features were systematically manipulated and controlled. However, investigating what stimulus features determine one’s disembedding abilities might be particularly potent in relation to individuals with ASD, as previous research has revealed the particular effect that differences in stimulus features can have, both in relation to EFT as well as in relation to other tasks, such as visual search or multiple object tracking (Almeida et al. 2010a, b, 2013; Evers et al. 2014; Van der Hallen et al. 2015, 2016). Without such investigation, it remains unclear what aspects of embedding drive the perceptual advantage sometimes observed (or inferred) in individuals with ASD. Is the advantage related to good continuation between target-lines and background-lines? Does the meaningfulness or three dimensionality present in some of the original EFT displays aid or hinder performance in individuals with ASD (and to what extent does this differ from what happens in TD individuals)? If individuals with ASD are less likely to see or interpret the embedding context as a meaningful whole, it may make it easier for them compared to TD individuals to locate the embedded target regardless of the context (Happé and Frith 2006; Mottron et al. 2006; Van der Hallen et al. 2015). A reduced tendency to see things in terms of their respective wholes, i.e., reduced global processing, may aid or push a more locally-oriented processing style, helpful in detecting search targets. Similar reasoning has been used to explain the fact that segmentation of block designs does not provide as great of an advantage to individuals with ASD compared to TD controls (Shah and Frith 1993).

The aim of the current study was to investigate disembedding in children with and without ASD, using the newly developed L-EFT as well as the M-EFT and D-EFT, evaluating the impact of meaningfulness and three dimensionality in relation to disembedding. Therefore, we administered the L-EFT, M-EFT and D-EFT to a group of children, aged 8–15 years, with ASD as well as a matched group of TD children. Previous research with the L-EFT, M-EFT and D-EFT in TD adults has revealed that good continuation and symmetry affect disembedding performance (i.e., lower accuracy, slower response times), and overall performance is influenced by the structure of the embedding context (i.e., better performance for 3D or meaningful trials compared to 2D or non-meaningful trials) (Chamberlain et al. 2017; de-Wit et al. 2017). Based on these results and all beforementioned EFT research in individuals with ASD, several predictions were formulated. Across both groups, it was predicted that (1) overall performance would be best for the L-EFT, followed by the M-EFT and then D-EFT, (2) L-EFT performance would decrease per increase in the number of target-line continuations, and (3) M-EFT and D-EFT performance would prove strongest for meaningfulness or 3D contexts, compared to matched, non-structured embedding contexts, i.e., it would prove easier to locate the target when one can easily grasp the gist or identify the structure compared to finding a similar target in an equally complex structure that is non-meaningful or 3D. Between groups, it was predicted that (1) L-EFT, M-EFT and D-EFT accuracy rates would be higher for the ASD group compared to the TD group, indicating stronger disembedding abilities in ASD, and (2) M-EFT and D-EFT performance would reveal a reduced effect of trial condition (i.e., meaningful vs. non-meaningful and 2D vs. 3D) for the ASD group compared to the TD group, indicating strong disembedding abilities in ASD irrespective of the embedding context or whole, as a result of a more locally-oriented processing strategy.

## Methods

### Participants

The research protocol was administered to two groups of 8-to-15-year old children. All participants were Dutch-speaking and reported normal or corrected-to-normal vision. Demographic details of both groups, ASD and TD, can be found in Table 1.

**Table 1:** Participant Characteristics

	ASD (17M:4F)		TD (13M:8F)		Two-sided *t* test
	M	SD	M	SD	*p* value
Age	12.54	1.64	11.71	1.26	0.07
Verbal IQ	99	14.65	98	13.80	0.77
Performance IQ	105	15.47	101	11.46	0.40
SRS	80.05	15.46	53.05	12.19	< .0001
ADOS	9.67	2.37			

*Note SRS data of one participant with ASD and of two TD participants is missing

The experimental group consisted of children with a formal clinical diagnosis of ASD, which were diagnosed according to DSM-IV-TR criteria (American Psychiatric Association 2000) by a multidisciplinary team. Recruitment was set up via the Autism Expertise Centre of the University Hospitals in Leuven. ASD diagnoses were re-evaluated within the research protocol using the Dutch version of the ADOS-2 conducted by a trained clinical psychologist (Gotham et al. 2006; Dutch version:; de Bildt et al. 2009). ASD diagnoses were re-confirmed for all children with the new ADOS algorithm for DSM-IV/ICD-10. The comparison group consisted of TD children recruited via mainstream schools. Children with a first-degree family member with a developmental disorder or children with a known child psychiatric disorder (information gathered from parents) were excluded. Participants with and without ASD were group-wise matched based on intelligence, age and gender-ratio (see Table 1). Intellectual abilities for all participants were estimated by administering an abbreviated version (Sattler 2001) of the WISC-III-NL (Wechsler 1992). ASD symptoms were evaluated using the Dutch version of the SRS-2 (Roeyers et al. 2011).

### Apparatus and Stimuli

All stimuli were created using an open source drawing program (Scribus Open Source Desktop Publishing). For more details on the stimulus design, see de-Wit et al. (2017). Stimulus presentation and response registration were controlled using custom software written in C# developed in Visual Studio. All tasks were performed on a set of identical Dell Inspiron desktop computers with a 23-inch monitor.

The L-EFT consists of 64 3AFC-trials in which the participant is asked to find a pre-defined target. Each trial presents one of 16 unique target shapes, four types of context patterns presented for each target shape. All target shapes are simple geometric figures, differing in the number of line segments used for the shape (3, 4, 6 and 8 line segments), as well as whether the target shape was symmetric vs. non-symmetric around its vertical axis, and formed an open vs. closed shape. The number of target lines that continues into the embedding context varies per target shape across its four trials, from 0 lines to a maximum of lines equal to the number of target lines (for more details, see de-Wit et al. 2017).

The M-EFT consists of 64 trials and was constructed in a similar manner, following the same criteria and principles as the L-EFT. However, in addition to the manipulations as described for the L-EFT, the contexts within which participants must try and locate a target shape are either meaningful (they represent real objects) or non-meaningful (they represent nonsense objects composed of the same parts as the meaningful objects). Care was taken to match meaningful and non-meaningful trials for total number of lines used and for the number of lines crossing through and extending from the target shape (Fig. 1).

The D-EFT consists of 32 trials and was constructed in a similar manner, following the same criteria and principles as the L-EFT. However, in addition to the manipulations as described for the L-EFT, the contexts within which participants must try and locate a target shape are either completely 2D or represent 3D surfaces arranged in depth (e.g., parts of cubes and bricks). Again, care was taken to match 2D and 3D trials for total number of lines used and for the number of lines crossing through and extending from the target shape (Fig. 2).

### Procedure

This study was approved by the ethical committee of the university hospitals UPC-KU Leuven and was incorporated within a larger series of studies on visual perception in individuals with ASD. Parent consent and child assent for each participant were obtained prior to testing. Participants were tested in a quiet and darkened room. Viewing distance was approximately 57 cm. No monetary compensation was provided; however, a small present was provided and transportation costs were reimbursed.

All participants completed the L-EFT, M-EFT and D-EFT: participants completed the L-EFT first, while the order of the M-EFT and D-EFT was counterbalanced between participants. Each of the three tasks was followed by a break and one or two other unrelated tasks. Prior to starting with the actual test items, the participants completed an extensive step-by-step practice protocol with six practice trials in which feedback was provided. Unfortunately, due to technical issues, not all D-EFT trials were collected as planned and partial data for 4 participants was lost.

The L-EFT consisted of 64 trials that were presented in a randomized order. For each trial, a 3AFC matching-to-sample paradigm was used in which the participant was presented with the target (above) and three response options (below). Of these three response options, one contained the target, and two were distractor contexts (see Figs. 1, 2). Participants had to choose which context contained the target as quickly and accurately as possible by clicking on the response alternative using the computer mouse. The stimuli were presented on the screen until the participant gave a correct response (no time limit). If they provided a wrong answer, visual feedback was given on their performance (a red square was shown around the chosen, incorrect alternative) and they were prompted to give a new response until they provided the correct answer. This procedure was put in place to ensure that participants would be motivated to actively find the target shape prior to providing an answer, reducing the likelihood of participants randomly guessing to advance through the task. The location of the correct context was varied randomly from trial-to-trial. The procedures for the M-EFT and D-EFT were identical to that of the L-EFT, except for the fact that the D-EFT only comprised 32 trials instead of 64 trials.

### Data-Analysis

Data-analysis was conducted using the general statistical software package SAS, Version 9.4 of the SAS System for Windows (SAS University Edition 2013). All analyses were conducted on the accuracy and RT data of the first response only. An arc-sine transformation was performed on the mean accuracy rates. Subject was included as a random factor. Significance tests were conducted with a significance level of 5%. Post-hoc tests were Tukey–Kramer corrected.

## Results

### L-EFT

#### Speed-Accuracy Trade-Off

There was a moderate correlation between accuracy and reaction time for the L-EFT, *r*(39) = 0.372, *p* < .02, 95% CI [0.06–0.37]. Therefore, all analyses are performed on both accuracy rates and reaction time.

#### Accuracy

**Fig. 3:**
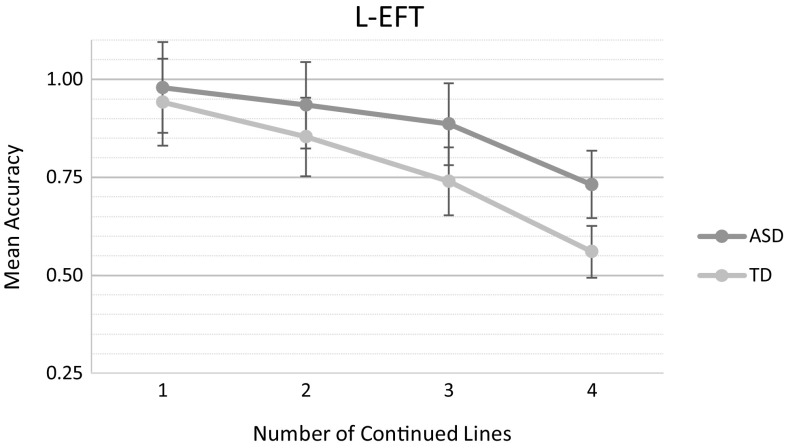
Mean accuracy by number of continued lines for both the ASD and TD participant group. Error bars represent standard error of the mean (SEM)

A 2 × 4 repeated-measures mixed model analysis with *Group* as between-subject variable, *Proportion of Continued Lines* as within-subject variable, and accuracy as dependent variable revealed a main effect of *Group, F*(1, 34) = 14.40, *p* = .0006, a main effect of *Proportion of Continued Lines, F*(3, 102) = 77.00, *p* < .0001, and a significant two-way interaction effect of *Group* x *Proportion of Continued Lines, F*(3, 102) = 3.35, *p* = .02. Overall, the ASD group performed more accurately than the TD group (ASD: *M* = 0.88, *SD* = 0.20; TD: *M* = 0.77, *SD* = 0.25). Post-hoc Tukey–Kramer analyses revealed that, while performance of both groups decreased with an increased number of continued lines, differences between both groups, in favor of the ASD group, proved significant in the case of 2 or more continued lines (see Fig. 3, t(102) = 3.67, *p* = .009; *t*(102) = 4.28, *p* = .001).

A 2 × 2 repeated-measures mixed model analysis with *Group* as between-subject variable, *Open vs. Closed Shape* as within-subject variable, and accuracy as dependent variable revealed a main effect of *Group, F*(1, 34) = 5.26, *p* = .03, in favor of the ASD group. No main effect of *Open vs. Closed Shape, F*(1, 34) = 0.01, *p* = .94, nor a two-way interaction effect of *Group* x *Open vs. Closed Shape, F*(1, 34) = 2.40, *p* = .13 was revealed.

A 2 × 2 repeated-measures mixed model analysis with *Group* as between-subject variable, *Symmetric vs. Non-symmetric Shape* as within-subject variable, and accuracy as dependent variable revealed a main effect of *Group, F*(1, 34) = 4.32, *p* = .05, in favor of the ASD group. No main effect of *Open vs. Closed Shape, F*(1, 34) = 1.18, *p* = .28, nor a two-way interaction effect of *Group* x *Open vs. Closed Shape, F*(1, 34) = 0.32, *p* = .58 was revealed.

#### RT

A 2 × 4 repeated-measures mixed model analysis with *Group* as between-subject variable, *Proportion of Continued Lines* as within-subject variable, and RT as dependent variable revealed a main effect of *Proportion of Continued Lines, F*(3, 102) = 101.15, *p* < .0001, and a significant two-way interaction effect of *Group* x *Proportion of Continued Lines, F*(3, 102) = 5.01, *p* = .003. No main effect of *Group, F*(1, 34) = 1.11, *p* = .30 was revealed. Post-hoc Tukey–Kramer analyses revealed no differences between both groups in the case of 0, 1 or 2 continued lines (*ps* > 0.98), while the ASD group proved marginally significantly slower compared with the TD group in the case of 3 continued lines, *t*(102) = 3.11, *p* = .048.

A 2 × 2 repeated-measures mixed model analysis with *Group* as between-subject variable, *Open vs. Closed Shape* as within-subject variable, and RT as dependent variable revealed no main effect of *Group, F*(1, 34) = 0.97, *p* = .33, *Open vs. Closed Shape, F*(1, 34) = 1.70, *p* = .20, or interaction effect of *Group* x *Open vs. Closed Shape, F*(1, 34) = 0.02, *p* = .89.

A 2 × 2 repeated-measures mixed model analysis with *Group* as between-subject variable, *Symmetric vs. Non-symmetric Shape* as within-subject variable, and RT as dependent variable also revealed no main effect of *Group, F*(1, 34) = 0.97, *p* = .33, *Open vs. Closed Shape, F*(1, 34) = 4.20, *p* = .05, or interaction effect of *Group* x *Open vs. Closed Shape, F*(1, 34) = 0.09, *p* = .76.

### M-EFT

#### Speed-Accuracy Trade-Off

There was a strong speed-accuracy trade-off, *r*(39) = 0.67, *p* < .0001, 95% CI [0.45, 0.81]. Because a high error rate (PC < 0.90) precludes the use of the inverse efficiency score (Bruyer and Brysbaert 2011), both accuracy and reaction time were submitted for further analysis.

#### Accuracy

**Fig. 4:**
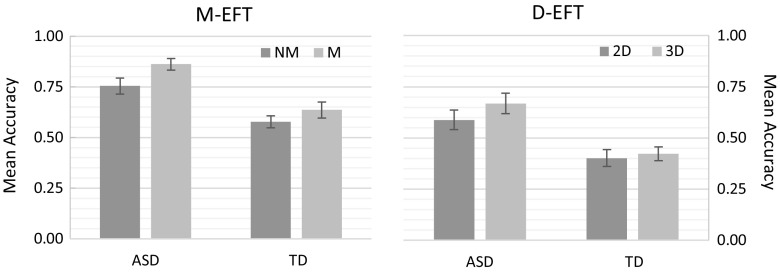
Mean accuracy for the non-meaningful (NM) and meaningful (M) condition of the M-EFT and two-dimensional (2D) and three-dimensional (3D) condition of the D-EFT task for the ASD and TD group. Error bars represent standard error of the mean (SEM)

A 2 × 2 repeated-measures mixed model analysis with *Group* as between-subject variable, *Condition* as within-subject variable, and accuracy as dependent variable revealed a main effect of *Group, F*(1, 35) = 20.53, *p* < .0001 and *Condition, F*(1, 35) = 31.73, *p* < .0001, as well as a significant two-way interaction effect of *Group* x *Condition, F*(1, 35) = 5.40, *p* = .03. Overall, the ASD group performed more accurately than the TD group (ASD: *M* = 0.81, *SD* = 0.15; TD: *M* = 0.61, *SD* = 0.15). Both participant groups performed more accurately on meaningful trials compared to non-meaningful trials, although this pattern of results was more pronounced for the ASD group compared to the TD group (see Fig. 4).

#### RT

A 2 × 2 repeated-measures mixed model analysis with *Group* as between-subject variable, *Condition* as within-subject variable, and RT as dependent variable revealed a main effect of *Group, F*(1, 35) = 5.16, *p* < .03. No main effect of *Condition, F*(1, 35) = 4.02, *p* = .06, nor a two-way interaction effect of *Group* x *Condition, F*(1, 35) = 3.17, *p* = .08 was revealed. On average, the ASD group was about 400 ms slower than the TD group (ASD: *M* = 3989, *SD* = 515; TD: *M* = 3561, *SD* = 671).

### D-EFT

#### Speed-Accuracy Trade-Off

There was a strong speed-accuracy trade-off, *r*(38) = 0.65, *p* < .0001, 95% CI [0.41, 0.80]. Because a high error rate (PC < 0.90) precludes the use of the inverse efficiency score (Bruyer and Brysbaert 2011), both accuracy and reaction time were submitted for further analysis.

#### Accuracy

A 2 × 2 repeated-measures mixed model analysis with *Group* as between-subject variable, *Condition* as within-subject variable, and accuracy as dependent variable revealed a main effect of *Group, F*(1, 30) = 10.09, *p* = .003. No main effect of *Condition, F*(1, 30) = 3.43, *p* = .07, nor a two-way interaction effect of *Group* x *Condition, F*(1, 30) = 1.33, *p* = .26 was revealed. The ASD group performed more accurately than the TD group (ASD: *M* = 0.63, *SD* = 0.21; TD: *M* = 0.41, *SD* = 0.15, see Fig. 4).

#### RT

A 2 × 2 repeated-measures mixed model analysis with *Group* as between-subject variable, *Condition* as within-subject variable, and RT as dependent variable revealed a main effect of *Group, F*(1, 30) = 6.11, *p* < .02. No main effect of *Condition, F*(1, 30) = 0.00, *p* = .99, nor a two-way interaction effect of *Group* x *Condition, F*(1, 30) = 0.49, *p* = .49 was revealed. On average, the ASD group was about 700 ms slower than the TD group (ASD: *M* = 4277, *SD* = 930; TD: *M* = 3582, *SD* = 974).

### Reliability

To evaluate the reliability of each EFT, split-half correlations were calculated (Spearman–Brown correction applied) across conditions and groups. Reliability results are *ρ* = 0.76 for the L-EFT, *ρ* = 0.93 for the M-EFT and *ρ* = 0.88 for the D-EFT, suggesting all three tasks show adequate reliability.

## Discussion

In the current study, we aimed to investigate disembedding in children with and without ASD, using the newly developed L-EFT, M-EFT and D-EFT, controlling for the number of continued lines and evaluating the impact of meaningfulness and dimensionality in relation to disembedding. First of all, the results revealed superior performance for the ASD group compared to the TD group for all three embedded figure tasks. Regardless of the type of EFT context, participants with ASD were more accurate at identifying the target than the TD group. In the easier L-EFT, the group difference was around 10%; in the more difficult M-EFT and D-EFT the group difference was around 20%, both times in favor of the ASD group. Noteworthy however, the ASD group was somewhat slower than the TD group for both the M-EFT and D-EFT task. Secondly, while performance of both groups decreased when the number of continued target-lines increased, the increase in number of continued target-lines proved more of a hindrance to the TD group than the ASD group. The ASD group performed more accurately than the TD group in the case of 2 or more continued lines, while RT differences, with the ASD group performing slower than the TD group, were only significant in the case of 3 continued lines. Finally, while both groups performed more accurately on meaningful trials compared to non-meaningful trials, this pattern of results was more pronounced for the ASD group compared to the TD group. No such difference, nor any effects opposite this finding, were found in relation to RT. Taken together, these results reveal superior performance for the ASD group compared to the TD group, for all three embedded figure tasks, although some main effects on accuracy might be, in part, related to main effects in RT, and the result of a difference in the speed-accuracy trade-off.

Superior disembedding performance in individuals ASD has long been argued for. Ever since Shah and Frith’s (1983) first report of atypical perceptual organization in individuals with ASD, which revealed children with ASD were better at detecting the embedded target shapes than controls, researchers have tried to replicate or explain these findings. A recent meta-analysis by Van der Hallen et al. (2015) evaluated all existing EFT data in individuals with ASD and, to some surprise, found no overall group difference between individuals with ASD and TD individuals; not in terms of accuracy rates, nor in terms of RT. However, a meta-analysis by Muth et al. (2014), less rigorously controlled as that by Van der Hallen et al., did find a small, significant difference in EFT performance in favor of individuals with ASD (*d* = 0.26). Interestingly, Muth et al. identified the initial study by Shah and Frith as one of few outliers, revealing a larger than typical difference in favor of the participants with ASD. Both meta-analyses, however, agreed on the fact that their overall patterns of results were clouded by substantially large heterogeneity between studies, and both studies were unable to identify significant moderators of the effect (or lack thereof), such as the dependent variable (RT or accuracy) or participants age, gender or level of intelligence.

Interestingly, the current study, which used EFT tasks with improved stimulus control, found superior disembedding for individuals with ASD for all three tasks, regardless of the embedding context. Whether participants were presented with standard EFT contexts, meaningful vs. non-meaningful contexts or 2D vs. 3D contexts, participants with ASD were more accurate in finding the predefined targets compared to the TD participants. In addition to that, the number of target lines continuing into the context proved less of a hindrance for the ASD group compared to the TD group, in line with a more locally-oriented processing style. However, the ASD group took more time to find the predefined target, at least in the case of the M-EFT and D-EFT. Taken together, these findings provide evidence to support the notion of superior disembedding in ASD. The heterogeneity in previous EFT data in mind, these results suggest that the type of embedding context might not play a significant part, at least when context is manipulated while all other factors are controlled for (i.e., total number of lines, number of lines running through the target shape, type of targets, etc.). Rather, these results suggest the heterogeneity is due to other between-study differences, such as the memory load, the required executive functions, or participant characteristics (Huygelier et al. 2018). However, while care was taken to try and mitigate the visual differences between the meaningful and non-meaningful contexts and the 2D and 3D contexts, we cannot rule out that any still uncontrolled factors, for instance the distribution of shapes in the contexts, may have had an effect on performance. Future research should attempt to control for any more remaining confounds to further help pinpoint any differences in performance between these different conditions.

While 3D contexts, as used in the D-EFT, had not been administered to individuals with ASD before, previous research by Brian and Bryson (1996) did investigate disembedding in individuals with ASD using both meaningful and non-meaningful contexts. Contrary to our findings, however, their results suggested meaningful contexts to be more difficult than non-meaningful context for both the ASD and TD group. Unfortunately, Brian and Bryson did not control for the stimulus features of their stimuli in the same way as was done for the L-EFT, M-EFT and D-EFT task. The difference in results, however, remains puzzling, as explanations for either findings have somewhat of an intuitive character to them. The results by Brian and Bryson, suggesting meaningful contexts are more difficult than non-meaningful contexts, would suggest that when a target is embedded in a known structure, the structure is identified in terms of the whole it represents, not the constituent parts, making it more difficult to identify substructures and, as a result, more difficult to find the target. However, our M-EFT and D-EFT results, indicating that strong contexts (i.e., meaningful contexts or 3D contexts) are easier than non-meaningful contexts, suggest it is easier to grasp the whole of the structure when the structure is meaningful or 3D, and then continue with a target-search, than is the case when the structure is equally complex but non-meaningful or 3D. This means that performance on the EFT represents more than the mere ability to ignore the global context, but also represents the ability of an individual to identify clues or strategies within the global context that will enable them to quickly identify the target. As suggested by Chamberlain and colleagues (2017), this would indicate that *embedding* occurs before *disembedding*: That is, organization of the context occurs before the individual constituents are processed and retrieved, reaffirming the primacy of global perceptual processing (Navon 1977). The fact that the ASD group was generally more accurate than the TD group and was generally less affected by the type of context, suggests that the ASD group is less influenced by the type or way a target is embedded and is better at disembedding in general.

What do these findings imply with regard to arguments on local vs. global perceptual organization in ASD? On the one hand, it seems simple: For all three EFT tasks used in the current study, superior performance for the ASD group compared to the TD group was revealed. This is particularly striking given the large heterogeneity in findings that have been revealed for EFT data in general. Regardless of the particular type of embedding context, when the stimulus features are controlled for, all three tasks yield similar group differences comparing individuals with ASD to TD individuals, suggestive of a stronger disembedding ability for individuals with ASD—or, as has been argued, strong local processing abilities (i.e., accuracy in finding a target). However, the ASD group had longer RTs than the TD group for both the M-EFT and D-EFT, suggesting a difference in speed-accuracy trade-off might be at play. Strong performance on the EFT has long been interpreted, not just as a reflection of disembedding abilities or field-(in)dependent cognitive styles, but also in relation to weak central coherence or enhanced local processing, especially with regard to individuals with ASD (e.g., Jolliffe and Baron-Cohen 1997; Mottron et al. 2006; Ring et al. 1999; Shah and Frith 1983). On the other hand, however, we have discussed how more and more research seems to indicate that disembedding, typically measured by the EFT, constitutes an independent factor or ability (e.g., Milne and Szczerbinski 2009), and very little variance is shared with other so-called local–global tasks. Along those lines, recent studies (Chamberlain et al. 2017; de-Wit et al. 2017; Huygelier et al. 2018) evaluated to what extent L-EFT performance specifically, could be predicted by estimates of local/global perceptual styles, executive functions and general intelligence and compared L-EFT performance to the original Group-EFT (G-EFT; Witkin et al. 1971) to directly contrast the construct validity of both tasks, Taken as a whole, the results of these studies imply that disembedding performance is consistent across different forms of the EFT and represents an independent perceptual process separate from those involved in similar local–global tasks, intelligence, or executive functioning. Moreover, their results showed that inter-task correlations within the EFT were high but low between the EFT and the Navon task. Also, performance on the L-EFT and G-EFT transferred very little to other tasks, as the amount of variance in performance in the L-EFT and G-EFT explained by differences in EF and intelligence was low, 15 and 25% respectively (Huygelier et al. 2018). In addition, the correlation between the L-EFT and the G-EFT was only moderate, suggesting critical differences between the new L-EFT and the previous G-EFT: the G-EFT proved more reliant on general task demands such as short term memory span than the updated and improved, more perceptual L-EFT. These results call into question the notion that EFT performance is representative of either a general perceptual or cognitive style, be it a tendency toward enhanced local perceptual processing, reduced global processing, weak central coherence or field independence, thereby supporting the factor analysis results of Milne and Szczerbinski (2009), who also suggested that disembedding was a discrete perceptual factor. Rather than being construed as a disadvantage, this should be considerate a strength for those wishing to study perceptual disembedding in isolation from more domain-general aspects of perceptual performance. This in mind, the results of the current study suggest superior perceptual disembedding in individuals with ASD – but make no claims regarding other local–global visual processing abilities that go beyond that.

## _Conclusion_

In sum, the current study aimed to investigate disembedding in children with and without ASD, using the newly developed L-EFT, as well as the M-EFT and D-EFT, evaluating the impact of meaningfulness and three dimensionality in relation to disembedding. The results revealed overall superior performance for the ASD group compared to the TD group, for all three embedded figure tasks. Regardless of the type of EFT context, participants with ASD performed more accurately than their TD counterparts. However, the ASD group took longer finding the predefined targets in both the M-EFT and D-EFT, suggestive of a difference in speed-accuracy trade-off.
